# Laser-Based Photobiomodulation for Orthodontic Pain: Mechanistic Evidence from Experimental Tooth-Movement Models

**DOI:** 10.3390/ijms27125519

**Published:** 2026-06-18

**Authors:** Ryo Kunimatsu, Kanoko Okazaki, Ayaka Nakatani, Kotaro Tanimoto

**Affiliations:** Department of Orthodontics and Craniofacial Developmental Biology, Graduate School of Biomedical and Health Sciences, Hiroshima University, 1-2-3 Kasumi, Minami-ku, Hiroshima 734-8553, Japananakatan@hiroshima-u.ac.jp (A.N.)

**Keywords:** photobiomodulation therapy, orthodontic pain, trigeminal nociception, neuron–glia interaction, neuroinflammation, near-infrared laser

## Abstract

Orthodontic pain, a fundamental biological response to mechanically induced tooth movement, is primarily associated with sterile inflammation and neurogenic processes within the periodontal ligament (PDL). Although photobiomodulation therapy (PBMT) has been widely investigated as a nonpharmacological approach for pain attenuation, its mechanisms of action remain incompletely understood, and current interpretations are often limited to peripheral anti-inflammatory effects. This review re-examines the biological basis of orthodontic pain by integrating evidence derived predominantly from in vitro and in vivo experimental studies. Particular emphasis is placed on neurogenic inflammation, neuropeptide regulation, and neuron–glia interactions along the trigeminal nociceptive pathway. PBMT can reduce periodontal inflammatory/neuropeptide-related markers and pain-related behaviors in selected models; however, evidence for direct central neuron–glia modulation remains largely marker-based and parameter-dependent. Direct functional validation of trigeminal circuit modulation (e.g., electrophysiological recordings or calcium imaging) remains limited in orthodontic pain models; thus, the proposed neuroimmune mechanisms should be interpreted as testable hypotheses for future work. By synthesizing mechanistic insights across multiple biological levels, this review proposes a broader framework for understanding PBMT-mediated pain modulation extending beyond conventional peripheral models. These perspectives may help clarify inconsistencies in the reported outcomes and provide a rationale for future hypothesis-driven experimental and translational research.

## 1. Introduction

### 1.1. Biological and Clinical Context of Orthodontic Treatment

Orthodontic treatment aims to improve oral function, occlusal stability, and facial esthetics through controlled modulation of craniofacial and dentoalveolar structures [1,2]. Beyond functional benefits, the correction of malocclusion contributes to psychosocial well-being and long-term oral health by reducing the risks associated with impaired mastication, oral hygiene challenges, and periodontal imbalance [1,2]. These therapeutic goals are primarily achieved through three-dimensional tooth movement using fixed orthodontic appliances, which impose sustained mechanical forces on the periodontal tissues.

Although orthodontic treatment is fundamentally a regenerative and adaptive biological process, it inevitably involves mechanical perturbation of the periodontal ligament (PDL) and surrounding alveolar bone. This biomechanical intervention triggers a cascade of cellular, vascular, and molecular responses that underlie both tissue remodeling and pain generation.

### 1.2. Orthodontic Tooth Movement

Although the teeth are embedded within the alveolar bone, they are not directly attached to the bone. Instead, they are suspended and supported by the PDL. The width of the human PDL is 0.15–0.38 mm, and it contains various cellular components, including osteoblasts, fibroblasts, osteoclasts, macrophages, and epithelial remnants of Malassez. In addition, the tissue includes collagen and oxytalan fibers as structural components, along with blood vessels, lymphatic vessels, and nerves.

Compared with other general tissues, the PDL is highly metabolically active, with a turnover rate 5–15 times greater than that of the gingiva and skin [3]. This unique tissue plays a central role in orthodontic tooth movement by maintaining homeostasis. Orthodontic tooth movement is driven by a series of biological processes involving adaptation of the alveolar bone to applied mechanical (orthodontic) forces, while inducing reversible changes within the PDL [4]. The alveolar bone and PDL generate different environments on the compression and traction sides of the tooth root under applied orthodontic forces. Blood flow is impaired on the compression side, whereas it increases on the traction side. Changes in the blood flow stimulate the release of bioactive substances such as prostaglandin (PG) E2 and inflammatory cytokines (interleukin [IL]-1β, tumor necrosis factor [TNF]-α), which significantly alter the oxygen tension and local chemical environment [5,6,7]. On the compression side, cyclooxygenase (COX)-2 levels are elevated, catalyzing the formation of prostaglandins from arachidonic acid [7]. Prostaglandins act on osteoclasts, increasing the intracellular cyclic adenosine monophosphate levels and enhancing the resorptive activity of osteoclasts [8]. Cytokines IL-1β, TNF-α, and PGE2 stimulate osteoblast differentiation and trigger the expression of receptor activator of nuclear factor kappa beta ligand (RANKL) and osteoprotegerin (OPG) [7,8]. Increased RANKL and macrophage colony-stimulating factor (M-CSF), along with decreased OPG, promote osteoclast differentiation and bone resorption. The compressed bone and PDL are degraded, after which the periodontium is reconstructed [5,6]. In contrast, alveolar bone deposition predominates on the traction side, resulting in increased osteoblast number and activity. The applied tensile force stimulates the proliferation of osteoblast precursors in the PDL and activates endothelial nitric oxide synthase to generate nitric oxide, which is involved in osteogenesis [9]. IL-10, an anti-inflammatory cytokine, also increases on the traction side, enhancing OPG production and reducing RANKL. Consequently, RANKL signaling is reduced, and bone deposition is promoted by inhibiting osteoclast formation, activity, and survival [7]. In addition, transforming growth factor (TGF)-β is elevated on the traction side, promoting PDL cell proliferation and chemotaxis, type 1 collagen gene expression, and osteoblast differentiation [10]. Matrix metalloproteinases (MMPs) and tissue inhibitors of metalloproteinases (TIMPs) are also upregulated, contributing to remodeling [11,12].

Furthermore, mechanical forces alter the neural components of the PDL. Tooth migration distorts nerve endings, releasing vasoactive neuropeptides, such as calcitonin gene-related peptide (CGRP) and substance P, which interact with the vascular endothelial cells [13]. Activated endothelial cells recruit circulating leukocytes, monocytes, and macrophages and contribute to the inflammatory responses within the PDL [14,15,16]. CGRP expression increases in response to the traction forces. CGRP acts as a vasodilator and stimulates plasma extravasation and leukocyte migration [17], further stimulating osteoblast activity while inhibiting osteoclasts [18]. Consequently, the number of osteoblasts increases, whereas the number of osteoclasts decreases, and bone generation and fiber remodeling of the PDL occur on the traction side. These mechanisms suggest that orthodontic forces induce bone resorption on the compression side and bone formation on the traction side. Although substantial progress has been made in elucidating the mechanisms underlying orthodontic tooth movement, several aspects remain incompletely understood, warranting further investigation.

### 1.3. Neuroinflammatory Mechanisms of Orthodontic Pain: From Periodontal Tissue Responses to Trigeminal Regulation

Orthodontic pain is not merely a secondary consequence of tissue remodeling but a complex neurobiological response emerging from interactions among inflammatory mediators, sensory neurons, and non-neuronal regulatory cells [19,20]. While sterile inflammation within periodontal tissues has traditionally been considered the principal driver of orthodontic pain, growing evidence supports a significant contribution of neurogenic mechanisms and trigeminal nociceptive processing in pain initiation and persistence [21,22].

From a mechanistic perspective, orthodontic pain can be interpreted as a multifactorial phenomenon arising from the dynamic interplay between inflammatory and neural regulatory processes. Nociceptive signals generated in the periodontal structures are conveyed through the trigeminal system and ultimately integrated within higher central nervous system (CNS) centers. For conceptual clarity, these mechanisms may be broadly categorized into peripheral and central components, reflecting distinct yet tightly interconnected biological pathways.

### 1.4. Mechanisms of Pain Generation by Orthodontic Forces in the Peripheral Tissues

Orthodontic forces trigger inflammatory responses in the periodontium. The inflammatory response involves three main components—blood vessels, cells, and chemical reactions—which interact to form a coordinated network. First, when orthodontic forces are applied to the teeth, blood vessels in the compressed area constrict, causing local ischemia [23]. Focal ischemia promotes anaerobic metabolism in the periodontal cells, leading to local acidosis. Local acidic conditions are detected by acid-sensing ion channel (ASIC) 3, contributing to pain perception. ASIC3, a proton-sensitive ion channel, is expressed in the periodontal sensory terminals [24]. Under acidic microenvironmental conditions, increased local H^+^ concentrations activate ASIC3 in the periodontal sensory endings, thereby triggering nociceptive signaling [25]. These nociceptive signals are transmitted to the trigeminal neurons in the trigeminal ganglion, leading to the release of neural mediators, including CGRP and substance P, both centrally (trigeminal nucleus) and peripherally (periodontium) [26,27,28,29]. These neurogenic mediators induce local vasodilation and enhance local inflammation [30], promoting CGRP expression in the sensory terminals of the trigeminal nerve and creating a positive feedback loop that amplifies pain [29]. These mediators also stimulate PGE2 production, which further sensitizes the sensory nerve endings, enhancing pain perception [31,32]. The ischemic, acidic microenvironment activates periodontal endothelial cells and fibroblasts to release NO, which further enhances vascular permeability in the periodontal tissues [33,34].

Increased vascular permeability causes leukocytes, such as neutrophils, monocytes, and lymphocytes, to accumulate in the periodontium [35,36,37,38,39]. When activated, these cells release inflammatory mediators, chemokines, and cytokines, including IL-1, IL-6, PGE2, TNF-α, interferon (IFN)-γ, M-CSF, and vascular endothelial growth factor, which further amplify local inflammation and contribute to pain generation [40,41,42]. Collectively, these mediators orchestrate the initiation and amplification of local inflammation during the early stages of orthodontic pain. Subsequently, IL-1, IL-6, TNF-α, IFN-γ, and M-CSF stimulate the osteoblasts and osteoclasts involved in alveolar bone remodeling and tooth movement, resulting in enhanced local inflammation [43,44,45]. In addition, M-CSF stimulates the differentiation of monocytes into macrophages and the recruitment and differentiation of osteoclasts, further enhancing local inflammation and pain [42]. Thus, vascular, cellular, and biochemical components act closely to generate local inflammation, which is subsequently transmitted to central nociceptive pathways.

### 1.5. Neuron–Glia Interactions and Central Sensitization

Orthodontic forces are perceived as painful stimuli by the sensory nerve endings in the periodontium. These signals are ultimately transmitted to the somatosensory cortex via a hierarchical neuronal pathway involving primary, secondary, and tertiary neurons. Primary neurons are trigeminal neurons in the trigeminal ganglion that are pseudo-unipolar in structure, possessing both peripheral and central processes. Peripheral processes extend to the facial skin, periodontal tissue, and oral mucosa, forming sensory endings that detect pain, mechanosensation, and thermal sensations. In contrast, the central processes form synapses with the trigeminal nucleus caudalis, which contains second-order neurons in the medulla oblongata. The trigeminal nucleus caudalis extends craniocaudally almost entirely in the medulla oblongata. Axons enter the medulla oblongata and migrate caudally to form synapses with the trigeminal nucleus, which then sends fibers to form the trigeminothalamic tract. This tract ascends to form synapses with the ventroposterior nucleus of the thalamus. The thalamus sends fibers to many areas of the brain, including the hippocampus, amygdala, and insular and somatosensory cortices, where nociceptive information is integrated and perceived as pain.

Ultimately, patients experience pain owing to orthodontic tooth movement. At the level of the trigeminal ganglion, several molecular changes associated with orthodontic pain have been reported. After force-induced pain, purinergic receptor-3 (P2X3) and CGRP are activated [46,47]. These molecules alter the biological properties of the neurons in the trigeminal ganglion and increase neuronal excitability [48], which can lead to enhanced activation of the trigeminal nucleus, resulting in thermal and mechanical hyperalgesia [49,50]. This mechanism may explain the increased sensitivity experienced during mastication in orthodontic patients. The trigeminal subnucleus caudalis (Vc) relays nociceptive information from the maxillofacial region. The caudal subnucleus has a three-part laminar structure: the marginal, gelatinous, and magnocellular layers.

The marginal layer and substantia gelatinosa correspond to laminae I and II of the spinal dorsal horn and contain nociceptive neurons receiving input from ipsilateral trigeminal receptive fields. Neuronal-glial interactions involving the neurons, microglia, and astrocytes have recently been recognized as key contributors to experimental tooth movement (ETM) [51,52]. In the CNS, glial cells are involved in the initiation and maintenance of persistent pain conditions. Microglia serve as early responders to injury and contribute to pain development [52]. Microglia release proinflammatory cytokines and prostaglandins, which activate the astrocytes [53] that contribute to the maintenance and amplification of pain signaling [54]. Previous studies [51,55] have demonstrated that the microglia and astrocytes in the dorsal horn of the spinal cord are activated after injury and inflammation of the peripheral nerves and are involved in dysalgesia, such as allodynia and hyperalgesia, after nerve injury and inflammation [56]. Ionized calcium-binding adapter molecule-1 (Iba-1) is a calcium/actin-binding protein expressed in macrophages and microglia. Iba-1 expression is induced by the cytokines and interferons involved in inflammatory responses [57]. Glial fibrillary acidic protein (GFAP)—an intermediate filament protein [58]—is primarily expressed in the astrocytes and involved in several key CNS processes, such as cellular motility and blood–brain barrier assembly [59]. Thus, Iba-1 and GFAP are the primary markers of microglia and astrocytes, respectively. Collectively, these findings suggest that orthodontic pain is regulated by interactions between trigeminal neurons and glial cells at both peripheral and central levels. However, the detailed control mechanism of pain caused by orthodontic forces remains unclear.

Further studies investigating multi-level neuronal pathways are warranted to elucidate the pathways involved in orthodontic pain.

### 1.6. PBMT in the Context of Neuroinflammatory Pain Modulation

The recognition that orthodontic pain involves peripheral inflammatory responses and neural plasticity and neuron–glia interactions carries important therapeutic implications. Conventional interpretations of PBMT have primarily emphasized its anti-inflammatory and tissue-healing properties. However, accumulating neurobiological evidence suggests that PBMT may additionally influence neural regulatory mechanisms relevant to pain modulation. Experimental findings indicate that PBMT can alter neuronal excitability, neuropeptide dynamics, and glial activity—all of which are critical components of orthodontic pain biology. These findings suggest the possibility that PBMT-mediated analgesia may extend beyond peripheral tissue effects and involve modulation of neuroinflammatory and neuro-glial pathways within the trigeminal system [60,61,62].

PBMT, historically referred to as low-level laser therapy (LLLT) [63], has been extensively investigated as a nonpharmacological strategy for regulating biological processes and alleviating pain [64,65]. In contrast to high-power surgical lasers, PBMT utilizes low-intensity light designed to induce photochemical and photobiological responses rather than thermal tissue ablation. The mechanistic basis of PBMT is commonly attributed to light-induced modulation of cellular metabolism, mitochondrial function, and intracellular signaling pathways [66]. Within clinical medicine and dentistry, PBMT has gained considerable attention owing to its reported anti-inflammatory and analgesic effects [66,67,68,69]. Nevertheless, the biological mechanisms underlying PBMT-mediated analgesia remain incompletely characterized, and prevailing interpretations have frequently centered on peripheral anti-inflammatory effects.

Emerging experimental evidence, however, indicates that light-induced biological modulation may not be restricted to classical inflammatory pathways. Observed effects on neuronal activity, neuropeptide regulation, and glial responses suggest that PBMT may interact with the neural regulatory systems governing nociceptive processing. Such a framework is particularly relevant in orthodontic pain, where neurogenic inflammation, trigeminal signaling, and neuron–glia interactions represent fundamental mechanistic elements.

## 2. Literature Identification and Scope

We conducted targeted searches in PubMed (1995–2025) using keywords related to photobiomodulation (PBMT/PBM/LLLT; laser/LED) and orthodontic pain/experimental tooth movement/trigeminal neuroinflammation, supplemented by citation tracking of key articles. This narrative review prioritizes mechanistic in vitro and in vivo studies relevant to orthodontic pain biology and PBMT parameters. Given the narrative focus, we did not perform a formal systematic review, Preferred Reporting Items for Systematic reviews and Meta-Analyses (PRISMA) workflow, or risk-of-bias assessment. We included English-language original in vitro and in vivo experimental studies addressing PBMT/PBM mechanisms relevant to orthodontic pain biology and parameter dependence, together with selected clinical studies and reviews providing mechanistic or translational context; we excluded non-English records, studies without mechanistic relevance to orthodontic pain or tooth movement, and reports lacking sufficient methodological detail (e.g., irradiation parameters).

## 3. In Vitro Evidence

### 3.1. Cellular and Molecular Bases of PBMT-Mediated Pain Modulation

In vitro models provide a controlled framework for dissecting the primary biological responses induced by PBMT. By isolating specific cell types and stimuli relevant to orthodontic tooth movement, these systems enable mechanistic evaluation of how photostimulation may regulate inflammatory signaling, neuronal excitability, and neuroimmune interactions. This section summarizes in vitro evidence supporting PBMT-mediated modulation across two interconnected biological layers: (i) PBMT effects under orthodontic force–mimicking mechanical loading and (ii) PBMT effects on neuronal and glial cellular models.

### 3.2. PBMT Effects Under Orthodontic Force-Mimicking Mechanical Loading (PBMT Effects on Periodontal-Associated Cells)

In clinical orthodontics, although PBMT has attracted attention as a nonpharmacological approach for pain attenuation, its analgesic efficacy and underlying mechanisms remain incompletely defined. Pain assessment relies largely on behavioral and sensory outcomes; therefore, many in vitro laser studies have instead focused on surrogate endpoints such as cellular responses, metabolic alterations, and the expression of inflammatory mediators. Key studies are summarized in Table 1.

Tsuka et al. [70] examined the effects of centrifugation-induced mechanical loading (489 rpm, 20 min) and neodymium-doped yttrium aluminum garnet (Nd:YAG) laser irradiation on bone metabolism–related markers in human osteoblast-like cells. Nd:YAG irradiation at 2.0 W in the absence of mechanical loading increased alkaline phosphatase (ALP), RANKL, and OPG expression, together with an elevated RANKL/OPG ratio, suggesting activation of bone metabolism–associated signaling. Under combined conditions (centrifugation loading plus 2.0 W irradiation), ALP and RANKL were further upregulated and OPG was reduced compared with mechanical loading alone, resulting in a higher RANKL/OPG ratio. These findings suggest that Nd:YAG irradiation modulates osteoblast-lineage remodeling signals, particularly the RANKL/OPG balance, and may interact with mechanically driven osteogenic pathways.

In a subsequent study, Tsuka et al. [71] investigated human gingival fibroblasts exposed to centrifugation-induced loading (500 rpm, 20 min) with or without Er:YAG laser irradiation. Er:YAG irradiation increased the expression of COX-2, IL-1β, TNF-α, and bone morphogenetic proteins (BMP)-2 and -4 even in the absence of mechanical loading; these responses increased in an irradiation energy-dependent manner, with peak effects reported at 1.2 W. Mechanical loading alone increased COX-2 expression but exhibited minimal effects on IL-1β, TNF-α, BMP-2, or BMP-4, whereas the combination of loading and Er:YAG irradiation increased the expression of these mediators. These findings suggest that PBMT can engage coupled inflammatory–osteogenic signaling programs in fibroblast-lineage cells and may interact with mechanical stimulation to form periodontal-associated cellular responses.

Tabatabaei et al. [72] assessed diode laser irradiation (100 mW; 3 or 6 J/cm^2^) in human PDL cells under orthodontic force–mimicking conditions (10% tensile strain or 25 g/cm^2^ compression), focusing on molecules related to bone metabolism and inflammatory responses. Under compression, laser irradiation increased OPG and periostin (POSTN) expression, whereas under tension it increased RANKL and sclerostin (SOST), indicating that PBMT can influence force-side–specific cellular programs induced by mechanical loading. Taken together, these findings suggest that PBMT may differentially regulate compression- versus tension-associated signatures (OPG/POSTN vs. RANKL/SOST), supporting side-specific modulation of PDL mechanobiological programs.

Huang et al. [73] evaluated diode laser irradiation (500 mW; 5 or 10 J/cm^2^; 2.5 or 5 s) in human PDL cells under a tensile condition mimicking the tension side of orthodontic loading (−100 kPa). Under tension, laser irradiation significantly increased cell viability at day 7 and suppressed the expression of inflammatory markers, including inducible nitric oxide synthase (iNOS), COX-2, and IL-1. In addition, osteocalcin activity, a late osteogenic marker, was significantly increased at day 7. These findings suggest that PBMT can shift tension-mimicking PDL cells toward a pro-survival, pro-osteogenic, and less inflammatory cellular phenotype.

Kao et al. [74] used a mouse cementoblast cell line (OCCM-30) subjected to negative pressure (tension) and examined the effects of CO_2_ laser irradiation (1.0 W; 3, 5, or 10 J/cm^2^; 3, 5, or 10 s) on cell viability and inflammatory marker expression. CO_2_ laser irradiation improved cell viability and supported cellular viability and function under tensile loading. IL-6 expression increased significantly at 3 and 5 J/cm^2^; iNOS peaked within 24 h and declined by day 7; COX-2 demonstrated a time-dependent increasing tendency. Although nociceptive endpoints were not assessed, the authors discussed the possibility that PBMT may support OCCM-30 survival and differentiation while modulating inflammatory responses, potentially contributing to tooth root repair and the attenuation of inflammatory root resorption during orthodontic treatment. Taken together, these findings suggest that CO_2_-based PBMT can preserve cementoblast viability under tensile stress while dynamically regulating stress-responsive inflammatory mediators, providing a plausible cellular basis for effects on repair–resorption balance rather than direct analgesia.

Collectively, these in vitro studies demonstrate that PBMT can modulate force-mimicking cellular programs in periodontal-associated cells, influencing key remodeling and inflammatory mediators such as the RANKL/OPG axis, COX-2–related pathways, osteogenic markers, and stress-responsive cytokine profiles. Importantly, the direction and magnitude of these effects vary across cell types (osteoblast-like cells, gingival fibroblasts, PDL cells, and cementoblasts), loading paradigms (compression vs. tension), and irradiation parameters, highlighting a major source of heterogeneity in the literature. This context dependence provides a mechanistic rationale for variable outcomes reported across orthodontic PBMT studies and underscores the need to extend mechanistic interrogation beyond periodontal tissues to neural and neuroimmune targets.

To date, in vitro PBMT studies explicitly combining compression-mimicking mechanical loading with periodontal/PDL-relevant cells remain limited. Most mechanobiology studies have characterized compression-side inflammatory and remodeling cues (e.g., COX-2/PGE2, RANKL/OPG, cytokine signaling), whereas PBMT-oriented in vitro studies have more often focused on tension paradigms or non-orthodontic inflammatory settings. Accordingly, leveraging established in vitro compression-side mechanobiology models—where key inflammatory and remodeling cues such as prostaglandin/cytokine signaling and osteoclastogenic programs are robustly induced—is essential for systematically interrogating how PBMT reshapes compression-driven pathways relevant to orthodontic pain and tissue remodeling. This gap also motivates parallel in vitro work in neuronal and glial models, given that compression-initiated peripheral inflammatory signals may ultimately converge on trigeminal neuroinflammatory processing. Accordingly, the next section focuses on in vitro evidence that PBMT can influence neuronal and glial cellular models relevant to trigeminal pain processing.

### 3.3. Effects of PBMT on Neuronal and Glial Cellular Models

Orthodontic pain is transmitted through the trigeminal pathways, where sensory ganglion neurons, their long neurites, and synaptic terminals constitute critical anatomical substrates for nociceptive transmission. In parallel, non-neuronal cells, including microglia and astrocytes, actively regulate neuroinflammatory signaling and can shape central sensitization. Thus, in vitro neuronal and glial models provide a mechanistic platform to examine whether PBMT can modulate cellular processes relevant to neuroinflammation, neuronal integrity, and pain-related signaling. Here, “PBM” is used interchangeably with PBMT to denote experimental PBM irradiation. Key studies using these readouts are summarized in Table 2.

#### 3.3.1. Neuronal Models

Saito et al. [75] investigated a neuronal model using nerve growth factor-differentiated pheochromocytoma (PC12) cells exposed to diode laser irradiation under either short (40–60 s; 0.3–0.5 J/cm^2^) or prolonged conditions (90 s × 20 exposures; total 15.0 J/cm^2^), and evaluated structural and functional alterations. Short-duration irradiation induced rapid swelling of neurite terminals and was associated with a tendency toward reduced numbers of neurosecretory granules. In contrast, prolonged irradiation caused marked terminal swelling, accompanied by an apparent depletion of neurosecretory granules. Moreover, terminal swelling and disruption were associated with cytoskeletal remodeling, including structural changes in filamentous actin, raising the possibility of neurite degeneration or retraction under higher cumulative exposure. These findings suggest that PBMT parameters can influence neurite terminal integrity and secretory dynamics in neuronal-like cells, processes potentially relevant to neurotransmission, although direct nociceptive endpoints were not assessed in this in vitro model.

Cho et al. [76] cultured primary trigeminal ganglion neurons and demonstrated that 808 nm near-infrared PBM promoted neurite outgrowth at lower radiant exposures (1–2 J/cm^2^), whereas a higher exposure (10 J/cm^2^) was not effective. Notably, a pulsed mode around 10 Hz produced the most robust growth responses, underscoring dose- and frequency-dependent effects in trigeminal sensory neurons.

In a complementary neuronal stress model, da Silva Oliveira et al. [77] examined whether PBM could mitigate glucose-induced neurotoxicity in mouse neuroblastoma-derived Neuro2a cells. Neuro2a cells were exposed to high glucose (100 mM for 48 h) and subsequently treated with PBMT using a low-level indium gallium aluminum phosphide diode laser (660 nm; 30 mW; 1.6 J/cm^2^; 15 s per well), administered either once or once daily for three consecutive days. PBM increased cell viability and reduced indices of cytotoxicity, and repeated irradiation enhanced neurite outgrowth. At the signaling level, these effects were accompanied by increased protein kinase B (AKT) phosphorylation, whereas extracellular signal-regulated kinase signaling exhibited no clear change. Collectively, these findings suggest that PBMT can engage pro-survival and neuroplasticity-related pathways (e.g., AKT) in neuronal-like cells under metabolic stress, providing a plausible molecular basis for considering direct neuronal targets of PBMT beyond peripheral anti-inflammatory mechanisms.

Beyond neuronal survival and neurite outgrowth, PBM has also been reported to preserve synaptic integrity under excitotoxic stress. Hong et al. [78] treated primary rat hippocampal neurons with kainic acid and applied PBM using an 850 nm light-emitting diode (LED) (10 min; ~6 J/cm^2^) shortly after excitotoxic stimulation. PBM mitigated kainic acid–induced synapse loss, as indicated by restored postsynaptic density protein 95 (PSD95) puncta colocalized with microtubule-associated protein 2 and recovery of PSD95 protein levels. PBM also improved dendritic morphology metrics compared with kainic acid alone. Mechanistically, PBM increased neuroligin (Nlgn)-3 expression, and *Nlgn3* silencing by small interfering RNA attenuated PBM-associated improvements in synaptic readouts, implicating an *Nlgn3*-linked component in PBM-mediated synaptic protection.

Wavelength dependence was highlighted by Lee et al. [79], who assessed primary sensory ganglion neurons (geniculate ganglion neurons) under oxidative stress. Cells were exposed to H_2_O_2_-induced oxidative stress and irradiated once using identical fluence settings (energy density 1.6 J/cm^2^), with varying wavelengths (633 nm, ~780 nm, or 804 nm; 20 mW for 150 s in their in vitro setup). Under oxidative stress, PBM at 633 nm and the intermediate near-infrared wavelength significantly improved neuronal viability compared with no PBM, whereas 804 nm produced only a modest or non-significant change. These findings suggest that even under matched fluence, PBM outcomes can be strongly wavelength-dependent in sensory neuronal preparations, implying that spectral differences may represent an underappreciated source of variability across PBMT studies of neural function and pain-related biology (Table 2).

#### 3.3.2. Glial Models

Von Leden et al. [80] examined microglial responses using the BV2 microglial cell line and primary microglia exposed to 808 nm irradiation across a range of energy densities (0.2, 4, 10, and 30 J/cm^2^). Lower-to-moderate doses (0.2–10 J/cm^2^) induced anti-inflammatory–associated markers (e.g., CD206 and TIMP1), whereas higher doses (4–30 J/cm^2^) also induced a subset of pro-inflammatory markers. Taken together, these findings imply that PBMT can shift microglial phenotypes in an energy density–dependent manner and support a biphasic response pattern, emphasizing the importance of parameter optimization when interpreting PBMT-mediated neuroimmune modulation.

Beyond microglia alone, Wang et al. [81] provided mechanistic evidence that PBM can modulate microglia–astrocyte crosstalk relevant to neurotoxic glial activation. In vitro, primary microglia were driven toward a pro-inflammatory activation state using lipopolysaccharide + IFN-γ, and astrocytes were induced toward a neurotoxic phenotype using C1q + TNF-α + IL-1α. PBM was delivered immediately after induction (810 nm; 6 mW; 4.5 cm^2^; 8 min) and repeated twice daily. PBM reduced markers of microglial activation (including iNOS and inflammatory cytokine transcripts) and attenuated neurotoxic astrocytic signatures (including complement C3 and lipocalin-2 [Lcn2]-associated readouts). Mechanistically, the study implicated suppression of Lcn2/Janus kinase 2 (JAK2)–STAT3 signaling-mediated crosstalk, supported by pharmacologic JAK2–STAT3 inhibition and Lcn2 knockdown/augmentation experiments. Moreover, conditioned media from induced glia increased apoptosis-related outcomes in a motor neuron–like cellular assay, and this neurotoxicity was alleviated when PBM was incorporated into the induction paradigm. Collectively, these findings suggest that PBMT can modulate both glial activation states and intercellular signaling loops that amplify neurotoxic phenotypes, offering a mechanistic framework for PBMT effects on the neuroinflammatory processes linked to central sensitization.

Astrocyte-directed evidence was further provided by Feng et al. [82] using an oxygen–glucose deprivation (OGD) paradigm. Primary astrocytes were subjected to OGD and subsequently treated with PBM (808 nm; 2 min; 25 mW/cm^2^; fluence ~3 J/cm^2^**;** initiated 24 h after OGD). OGD increased neurotoxic/pro-inflammatory astrocytic markers (e.g., C3d) and elevated inflammatory mediators, including iNOS and IL-1β, whereas PBM significantly suppressed these OGD-induced changes. Taken together, these findings imply that PBMT can inhibit stress-induced neurotoxic astrocytic polarization and dampen pro-inflammatory mediator production in vitro, reinforcing the plausibility of astrocytic mechanisms as PBMT targets relevant to neuroinflammatory pain processing.

Additional astrocyte-level evidence suggests that PBM can influence glial growth-state biology under developmental culture conditions. In an astrocyte-dominant culture system, Yoon et al. [83] reported that 660 nm LED irradiation (10 mW/cm^2^ for 10 min; 6 J/cm^2^; delivered twice during days in vitro [DIV]7–DIV14) increased reactive oxygen species activity and selectively elevated astrocyte proliferation indices (bromodeoxyuridine+/GFAP+ and Ki67+/GFAP+) without overt changes in overall culture viability. These findings suggest that PBM can modulate astrocytic proliferative and maturation-state markers in a parameter- and context-dependent manner (Table 2).

#### 3.3.3. One-Sentence Integration

Across neuronal and glial in vitro systems, PBMT effects are strongly parameter-dependent (wavelength/irradiance/fluence and exposure schedule), providing a mechanistic basis for heterogeneity in reported PBMT outcomes and underscoring the need for orthodontic pain–relevant experimental designs that explicitly link neural/glial molecular readouts to trigeminal nociceptive function.

Building on this framework, the next section examines in vivo ETM models within the trigeminal system to determine whether PBMT attenuates nociceptive behaviors and suppresses neural–glial activation markers across peripheral and central trigeminal sites.

## 4. Effects of Laser Irradiation on Pain in ETM (In Vivo Studies)

In vivo ETM models provide a translational framework for testing whether PBMT-mediated cellular effects observed in vitro manifest as measurable analgesia and neuroinflammatory modulation under orthodontic loading. In these models, controlled orthodontic forces are applied (e.g., elastic separators or nickel–titanium coil springs) to induce periodontal sterile inflammation and trigger nociceptive input into the trigeminal system. ETM enables simultaneous evaluation of pain-related behaviors (e.g., facial grooming, vacuous chewing movements, or jaw-opening reflex thresholds) and molecular/cellular readouts across both peripheral and central sites. Peripheral assessments commonly include inflammatory mediators and neuropeptides within periodontal tissues (e.g., COX-2/PGE2, IL-1β, CGRP, and substance P), whereas central analyses focus on activity-dependent neuronal markers and glial responses within the trigeminal structures (e.g., Fos-related measures, Iba-1 for microglia, and GFAP for astrocytes) in the trigeminal ganglion and Vc. In the following section, we summarize mechanistic in vivo ETM studies of laser-based PBMT, emphasizing how irradiation parameters, timing, and target tissues relate to behavioral outcomes and trigeminal neuroimmune signatures.

Several in vivo studies have investigated whether laser irradiation attenuates pain associated with ETM. Key studies are summarized in Table 3. Seiryu et al. [84] inserted elastic separators between rat molars (Wald method) and applied CO_2_ laser irradiation to the gingiva, assessing Fos immunoreactivity in the Vc and examining periodontal tissues. CO_2_ laser irradiation suppressed ETM-induced Fos-IR in the Vc, a marker of early nociceptive activation. They also evaluated neuronal markers such as PGP 9.5 and CGRP immunoreactivity in the PDL tissues and reported that the maximal surface temperature during CO_2_ laser irradiation (1.0 W, pulsed, 30 s) remained below 40 °C [84].

Deguchi et al. [85] similarly inserted elastic separators and delivered CO_2_ laser irradiation to the gingiva (30 s; 0.01 s on/0.09 s off; 1.0 W). In the Vc, c-Fos expression peaked 1 day after ETM initiation and was significantly reduced in the CO_2_ laser- irradiated group compared with nonirradiated controls. In addition, glial markers increased at later time points: CD11b (microglial marker) and GFAP (astrocytic marker) were significantly elevated from days 3 to 7, and CO_2_ laser irradiation showed a trend toward suppressing these changes, although differences did not reach statistical significance.

Tsuchiya et al. [86] applied 50 g orthodontic force using nickel–titanium closed-coil springs and compared analgesic effects of CO_2_ laser irradiation (10,600 nm; 10.6 µm, 0.5 W, continuous wave; 30 s or 600 s) and diode laser irradiation (808 nm, 0.5 W, continuous wave; 30 s or 600 s). Pain-related function was assessed using the jaw-opening reflex (JOR), an objective readout of orofacial nociceptive processing. CO_2_ laser irradiation to the palatal mucosa increased the JOR threshold, indicating reduced reflex excitability; notably, a similar reduction was induced by topical anesthesia and thermal stimulation, whereas diode laser irradiation did not reproduce this effect. However, when either laser was applied for 30 s immediately after force application, both groups showed reduced trigeminal ganglion GFAP expression and decreased JOR excitability 1 day later. Collectively, these findings suggest that CO_2_ and diode laser irradiation can influence ETM-associated nociceptive processing, potentially via distinct mechanisms.

Nakatani et al. [87] evaluated analgesic effects of high-frequency pulsed near-infrared diode irradiation using an ETM model with nickel–titanium coil springs. After ETM induction, diode laser irradiation (910 nm; pulse width 200 ns; 40 kHz; 120 J/cm^2^) reduced nociceptive behaviors, including facial grooming frequency and vacuous chewing movement (VCM) duration. In parallel, ETM-induced increases in Fos-IR neurons within Vc laminae I/II were significantly suppressed, and ETM-associated increases in Iba-1 (microglia) and GFAP (astrocytes) immunoreactivity were also significantly reduced, suggesting attenuation of trigeminal neuroinflammatory activation.

Using the same ETM paradigm and irradiation conditions, Nakatani et al. [88] further examined the periodontal tissues and reported that ETM-induced increases in IL-1β, PGE_2_, COX-2, and CGRP protein expression on the compression side of the PDL were significantly suppressed by near-infrared diode irradiation. Similarly, Okazaki et al. [89] applied diode irradiation to the periodontal tissues (808 nm; pulse width 20 ms; 100.8 J/cm^2^) and found reductions in the ETM-evoked nociceptive behaviors (facial grooming and VCM). In addition, ETM-induced increases in SP, CGRP, and heat shock protein 70 on the compression side of the PDL were significantly suppressed. Taken together, these studies indicate that near-infrared diode laser irradiation can reduce peripheral inflammatory mediators and neuropeptides in the compression-side periodontal tissues, coinciding with attenuation of the trigeminal neuronal activity markers and glial activation, and thereby may alleviate ETM-associated pain.

Mechanistically, near-infrared PBMT is often discussed in the context of mitochondrial photoreception [90] (e.g., cytochrome c oxidase) and downstream redox-sensitive signaling that can modulate inflammatory transcriptional programs [91]. In the ETM models, such modulation may contribute to reduced COX-2/PGE_2_ and cytokine expression in the periodontal tissues, decreased peripheral nociceptive drive, and subsequent attenuation of neuronal activity and microglial/astrocytic responses within central trigeminal sites. In contrast, CO_2_ laser irradiation effects may involve superficial photothermal or anesthesia-like components, as suggested by JOR findings, underscoring the importance of distinguishing wavelength-specific mechanisms.

Future studies should systematically determine optimal irradiation parameters (wavelength, fluence/irradiance, pulse structure, treatment schedule, and target tissue) required for safe and reproducible analgesic effects. In addition, deeper mechanistic interrogation is warranted to link peripheral periodontal changes (cytokines, PGE_2_, CGRP/SP) with trigeminal circuit readouts, including neuronal excitability and neuron–glia interactions across trigeminal ganglion and Vc.

### 4.1. Mechanistic Considerations for PBMT-Mediated Analgesia

PBMT has been proposed to attenuate orthodontic pain through multiple, potentially overlapping mechanisms operating across peripheral and central levels. First, PBMT may attenuate sterile inflammation in the periodontal tissues, including pathways linked to COX-2/PGE_2_ and pro-inflammatory cytokines, thereby reducing nociceptor sensitization and peripheral nociceptive drive. Second, PBMT may modulate neuropeptide signaling and neuronal excitability within periodontal afferents and trigeminal ganglion neurons, consistent with in vitro observations of parameter-dependent effects on neurite terminal integrity, neurite outgrowth, and synaptic markers. Third, PBMT may suppress trigeminal neuroinflammation within central projection sites, as suggested by the reduced Fos-related neuronal activity and suppressed microglial/astrocytic activation markers (Iba-1 and GFAP) reported in ETM studies. Finally, although evidence remains limited, engagement of endogenous descending inhibitory pathways has been discussed and warrants hypothesis-driven investigation using circuit-level readouts. Importantly, CO_2_ irradiation may involve superficial photothermal or anesthesia-like components, whereas near-infrared diode protocols are more consistent with PBMT-type photochemical modulation; therefore, wavelength-specific mechanisms should be interpreted separately when integrating analgesic evidence.

Future research should also incorporate trigeminal ganglion neuron–satellite glial cell interactions and pain-proximal readouts (e.g., CGRP/substance P release, P2X3/TRPV1/ASIC signaling, and excitability measures) to bridge molecular changes with trigeminal nociceptive function.

A persistent limitation of orthodontic ETM studies is that trigeminal modulation is primarily inferred from behavioral proxies and molecular markers such as Fos-related measures, *Iba-1*, and *GFAP*, rather than from direct functional readouts. Functional studies in related sensory-neuron and peripheral-nerve models nevertheless support biological plausibility: 940 nm irradiation has been reported to modulate intracellular calcium influx in primary trigeminal ganglion neurons [92], and near-infrared (808 nm) irradiation of peripheral nerve selectively suppressed mechanically evoked nociceptive firing in lamina II of the rat spinal dorsal horn, persisting for hours without histological nerve damage [93]. Comparable selective inhibition of small-diameter nociceptive fibers without motor impairment has been described following direct photobiomodulation of peripheral nerve [94]. These findings should not be interpreted as direct functional validation within orthodontic ETM paradigms; rather, future ETM studies should integrate electrophysiology or calcium imaging with behavioral and molecular endpoints to establish causal links between PBMT, trigeminal excitability, and neuron–glia interactions.

Behavioral and reflex-based readouts used in ETM models (e.g., facial grooming, vacuous chewing movements, and jaw-opening reflex thresholds) provide useful proxies for orofacial nociceptive processing, but they do not directly quantify the subjective intensity or affective dimensions of human pain. Notably, the jaw-opening reflex can be evoked by both noxious and non-noxious stimuli and does not consistently correlate with the magnitude of perceived pain [95], and rodent orofacial pain assays in general face intrinsic translational limitations [96]. Reflex measures can additionally be influenced by non-analgesic factors, including thermal or anesthesia-like effects. These outcomes should therefore be interpreted as convergent indicators of nociceptive processing rather than direct equivalents of clinical VAS or NRS scores.

### 4.2. Parameter Dependence and Sources of Heterogeneity

PBMT effects are not uniformly beneficial and appear highly dependent on wavelength, fluence/irradiance, pulse structure, timing, and target tissue, consistent with a biphasic dose–response relationship (the Arndt–Schulz pattern) [97]. Lower fluences or specific pulse conditions may support neurite growth or anti-inflammatory phenotypes, whereas higher cumulative exposures can be ineffective or associated with pro-inflammatory markers or neurite terminal disruption, as seen in the in vitro studies summarized above. Critically, CO_2_ and near-infrared diode protocols should not be interpreted as mechanistically interchangeable. These parameter dependencies argue against assuming a universal PBMT effect and instead support standardized reporting of wavelength, irradiance, fluence, pulse structure, timing, and target tissue to reduce overgeneralization across heterogeneous protocols.

CO_2_ laser irradiation (10.6 μm) is strongly absorbed by tissue water and acts predominantly through superficial photothermal mechanisms, whereas near-infrared diode wavelengths penetrate more deeply and are more consistent with photochemical modulation via mitochondrial chromophores such as cytochrome c oxidase [98].

### 4.3. Negative, Null, and Parameter-Dependent Findings

A balanced appraisal requires acknowledging that PBMT does not uniformly reduce pain. In vitro, higher cumulative exposures have been associated with neurite terminal swelling and degenerative changes, and higher microglial doses have induced pro-inflammatory markers, illustrating biphasic, parameter-dependent behavior. Clinically, the evidence is heterogeneous: whereas several randomized trials report reduced orthodontic pain, others report no significant benefit—for example, a randomized trial of single-dose 940 nm diode photobiomodulation after initial archwire placement found no significant analgesic effect [99]. These observations argue against assuming a universal PBMT effect and reinforce that outcomes are strongly parameter-dependent.

### 4.4. Clinical Context

Clinical evidence for PBMT/low-level laser therapy in orthodontic pain is heterogeneous. A Cochrane review concluded that laser irradiation may reduce pain during orthodontic treatment in the short term, but the certainty of evidence was low [100]. More recent randomized trials also show variable results, including studies reporting no significant pain reduction after single-dose diode photobiomodulation [99]. These clinical inconsistencies are consistent with the experimental literature showing strong parameter dependence and support the need for standardized reporting and mechanistically informed trial design, rather than broad claims of uniform efficacy.

## 5. Conclusions

A graphical summary is provided (Graphical Abstract) to enable a concise overview of the multi-level mechanism proposed in this review. It highlights how orthodontic force induces peripheral sterile and neurogenic inflammation that propagates through trigeminal pathways and neuron–glia interactions, and how PBMT may modulate peripheral mediators, neuronal plasticity, and glial activation in a strongly parameter-dependent manner.

PBMT has been investigated as a noninvasive and clinically feasible adjunct for orthodontic pain management. However, its analgesic benefit appears strongly parameter-dependent, and the current clinical evidence remains heterogeneous. PBMT should therefore be regarded as a promising but optimization-dependent approach rather than a uniformly effective intervention. Its underlying analgesic mechanisms also remain incompletely understood. Future research should focus on standardized mechanobiology-informed experimental platforms, particularly compression-relevant paradigms. Further investigation into optimal irradiation parameters (e.g., wavelength, fluence, and exposure timing) is essential for safe and effective clinical application. Specifically, future studies should employ standardized in vitro compression models—for example, periodontal ligament cells or cementoblasts under defined static or cyclic compressive loading—combined with PBMT at systematically varied wavelength, fluence, and timing, and evaluated using both inflammatory/neuropeptide markers and functional readouts such as calcium imaging, to directly clarify compression-side mechanisms relevant to orthodontic pain.

In particular, photothermal CO_2_ and photochemical near-infrared diode modalities should be interpreted separately rather than as interchangeable approaches, as their distinct mechanisms of tissue interaction likely contribute to the heterogeneity of reported outcomes. Advances in mechanistic and high-quality clinical studies will be critical to translating PBMT into evidence-based orthodontic pain management [101,102]. Importantly, pain-related outcomes in experimental tooth movement models (e.g., facial grooming, vacuous chewing movements, and jaw-opening reflex thresholds) are proxy measures of nociception and may not fully recapitulate subjective pain experience in humans. Moreover, much of the mechanistic evidence in orthodontic PBMT research is derived from molecular markers (e.g., Fos-related measures, Iba-1, and GFAP) rather than direct functional readouts. Future studies integrating PBMT with electrophysiological recordings or calcium imaging across trigeminal ganglion and trigeminal subnucleus caudalis circuits will be essential to validate causal neuromodulatory mechanisms and to better define parameter-dependent null or adverse outcomes.

## Figures and Tables

**Table 1:** PBMT Effects Under Orthodontic Force–Mimicking Mechanical Loading (Periodontal-Associated Cells).

Study ID	Authors	Publication Year	Type of Laser Irradiation Protocol	Cell Type Loading Conditions	Major Finding
[70]	Tsuka et al.	2020	• 1064 nm	Human osteoblast-like cells	PBMT promoted the expression of ALP and RANKL, increased the RANKL/OPG ratio, and affected bone metabolism under conditions of mechanical stimulation.
			• Pulse	Centrifugal force of 489 rpm	
			• 5.17 J/cm^2^		
			0.6 W		
			• 17.23 J/cm^2^		
			2.0 W		
[71]	Tsuka et al.	2020	• 2940 nm	Human gingiva fibroblast cells	PBMT promoted the expression of COX-2, IL-1β, TNF-α, BMP-2, and BMP-4 under mechanical stimulation conditions, affecting bone metabolism.
			• Pulse	Spinning force of 500 rpm	
			• 1.875 J/cm^2^		
			0.6 W		
			• 3.13 J/cm^2^		
			1.0 W		
			• 3.75 J/cm^2^		
			1.2 W		
[72]	Tabatabaei et al.	2023	• 980 nm	Human periodontal ligament cells	PBMT promoted the expression of OPG and POSTN on the compression side, as well as the expression of RANKL and SOST on the tension side, affecting the cellular responses induced by orthodontic force.
			• Continuous	tensile force of 10% stretch	
			• 100 mW	compressive force of 25 g/cm^2^	
			• 3, 6 J/cm^2^		
			• 15, 30 s		
[73]	Huang et al.	2013	• 670 nm	Human periodontal ligament cells	PBMT increased cell viability.
			• Continuous	−100 kPa tension	It also suppressed the expression of iNOS, COX-2, and IL-1, and promoted osteocalcin activity on day 7.
			• 500 mW		
			• 5, 10 J/cm^2^		
			• 2.5, 5 s		
[74]	Kao et al.	2024	• 10.6 μm	Cementoblasts from mice	PBMT improved cell viability and promoted IL-6 expression.
			• Continuous	Negative pressure (tension)	iNOS reached its peak within 24 h, and subsequent expression was suppressed, but COX-2 expression was not suppressed.
			• 1.0 W		
			• 3, 5, 10 J/cm^2^		
			• 3, 5, 10 s		

**Table 2:** Effects of PBMT on neuronal and glial cellular models (In vitro).

Study ID	Authors	Publication Year	Type of Laser Irradiation Protocol	Cell Type Conditions	Major Finding
[75]	Saito et al.	2010	• GaAlAs diode laser	Nerve cell	Short-term laser irradiation caused swelling of some nerve terminal endings and a decrease in the number of nerve granules immediately after irradiation.
			• 0.3–0.5 J/cm^2^	Unrelated	With long-term laser irradiation, most terminal portions swelled significantly, and all nerve granules were released and disappeared.
			40–60 s		Additionally, F-Actin underwent structural changes, and the nerve processes degenerated and retracted.
			• 15.0 J/cm^2^		
			90 s		
			20 sessions		
[76]	Cho et al.	2020	• 808 nm diode laser	Primary trigeminal ganglion (TG) neurons	808 nm PBM promotes neurite outgrowth in primary trigeminal ganglion neurons in a strongly parameter-dependent manner, with lower fluences (1–2 J/cm^2^) and ~10 Hz conditions producing the most robust growth responses, whereas higher fluence (10 J/cm^2^) was less effective.
			• CW and PW (duty 50%)	neurite growth	
			• 500 mW		
			• PW frequency: 1/10/100 Hz		
			• Fluence: 1/2/10 J/cm^2^		
			• CW time: 40/80/400 s		
			• PW time: 80/160/800 s • Daily irradiation for 3 days		
[77]	Oliveira V.R.S. et al.	2020	• 660 nm InGaAlP diode laser	Mouse neuroblastoma-derived: Neuro2A (N2A) cells	In Neuro2A cells under high-glucose neurotoxicity, 660 nm PBM (1.6 J/cm^2^) improved viability and—after repeated exposures—enhanced neurite outgrowth with increased p-AKT, supporting a pro-survival/neuroplasticity mechanism.
			• 30 mW	High glucose load: Final 100 mM (48 h)	
			• Fluence: 1.6 J/cm^2^		
			• 15 s (spot 0.28 cm^2^)		
			• CW		
			• Irradiations: 1× or 3× (24 h		
			intervals)		
			• Irradiation from the bottom		
			of each well		
[78]	Hong N et al.	2023	• 850 nm LED	Primary rat hippocampal neurons excitotoxicity model induced by kainic acid (KA)	PBM suppresses KA-induced synaptic impairment and increases *Nlgn3*.
			• 16 mW/cm^2^		Furthermore, *Nlgn3* knockdown attenuated the PBM effect, suggesting the possibility of *Nlgn3*-dependent synaptic protection.
			• distance 4 cm		
			• 600 s		
			• 9.6 J/cm^2^		
			• Irradiation 30 min after KA treatment		
[79]	Lee JH et al.	2021	• 633/780/804 nm	Primary geniculate ganglion neurons (GGNs)	At the same fluence, 633 nm and 780 nm suppressed cell death under oxidative stress. On the other hand, 804 nm showed no suppressive effect.
			• Single irradiation	Oxidative stress was induced using H_2_O_2_ (100 µM, 24 h)	
			• 20 mW		
			• 150 s		
			• 1.6 J/cm^2^		
			• 1.9 cm^2^		
			• 10.5 mW/cm^2^		
[80]	Von Leden et al.	2013	• 808 nm	microglia cells from mice	PBMT promoted the expression of anti-inflammatory M2 markers (CD206, TIMP1). It also promoted the induction of some pro-inflammatory M1 markers and influenced the microglial phenotype in an energy density-dependent manner.
			• 50 mW	unrelated	
			• 0.2 J/cm^2^ 28 s		
			• 4 J/cm^2^ 565 s		
			• 10 J/cm^2^ 1413 s		
			• 30 J/cm^2^ 4239 s • 90 J/cm^2^ 12,717 s		
[81]	Wang et al.	2021	• 810 nm	Primary microglia/astrocytes	PBM suppressed inflammatory microglial activity (iNOS, etc.) and A1-like astrocyte-related genes (C3/Lcn2, etc.).
			• 6 mW	microglia: LPS (1 µg/mL) + IFN-γ (20 ng/mL)	Suppression of Lcn2–JAK2/STAT3 crosstalk was suggested as a potential mechanism.
			• area 4.5 cm^2^	astrocytes: C1q (400 ng/mL) + TNF-α (30 ng/mL) + IL-1α (3 ng/mL)	
			• 8 min		
			• Irradiation twice daily every 12 h, starting immediately after induction		
[82]	Feng Y et al.	2023	• 808 nm laser	in vitro OGD (oxygen–glucose deprivation) model	PBM supports neuronal survival/anti-apoptosis/synaptic protein retention after OGD, and reduces C3d, iNOS, and IL-1β in astrocytes.
			• 7-day repeated irradiation in vivo	Primary cortical neurons, N2a, primary cortical astrocytes	
			• single-dose PBM in vitro		
[83]	Yoon et al.	2021	• PBM	Astrocyte-dominant primary culture	ROS levels increased with 660 nm irradiation, while the BrdU/Ki67 positivity rate in astrocytes increased (proliferation promotion).
			• 660 nm LED		No significant adverse effects on overall viability were observed.
			• CW		
			• distance 4 cm,		
			• 10 mW/cm^2^		
			• 10 min,		
			• 6 J/cm^2^		
			• DIV7 and DIV10		

**Table 3:** Effects of Laser Irradiation on Pain in Experimental Tooth Movement (In vivo Studies).

Study ID	Authors	Publication Year	Type of Laser	Animals	Major Finding
			Irradiation Protocol	Methods of OTM	
[84]	Seiryu et al.	2010	• CO_2_ laser	Rats	CO_2_ laser irradiation to the gingiva just after tooth movement caused a significant decrease in Fos-IR neurons. PGP 9.5- and CGRP-positive nerve fibers were observed in the periodontal ligament (PDL) of all study groups.
			• Pulse	Elastic modules	
			• 1.0 W		
			• 30 s		
[85]	Deguchi et al.	2017	• CO_2_ laser	Rats	PBMT inhibited the expression of c-fos in the Vc region.
			• Pulse	elastic modules	Additionally, PBMT tended to inhibit the expression of CD-11b and GFAP.
			• 1.0 W		
			• 30 s		
[86]	Tsuchiya et al.	2020	• 10,600 nm;	Rats	CO_2_ laser irradiation increased the threshold for evoking the jaw-opening reflex, whereas semiconductor laser irradiation did not.
			808 nm	Nickel titanium closed coil spring	Additionally, both CO2 laser irradiation and semiconductor laser irradiation suppressed the expression of GFAP in the trigeminal ganglion.
			• Continuous		
			• 500 mW		
			• CO_2_ laser: 15, 30,		
			600 s		
			• Diode laser:		
			30, 600 s		
[87]	Nakatani et al.	2022	• 910 nm	Rats	PBMT suppressed the expression of Fos-IR, Iba-1, and GFAP in the Vc region, as well as nociceptive pain behavior in rats.
			• Pulse	Nickel titanium closed coil spring	
			• 300 mW		
			• 120 J/cm^2^		
			• 10 min		
[88]	Nakatani et al.	2023	• 910 nm	rat	PBMT suppressed the expression of IL-1β, PGE2, COX-2, and CGRP in the periodontal ligament region on the compression side.
			• Pulse	Nickel titanium closed coil spring	
			• 300 mW		
			• 120 J/cm^2^		
			• 10 min		
[89]	Okazaki et al.	2025	• 808 nm	Rats	PBMT suppressed the expression of CGRP, SP, and HSP 70 in the periodontal ligament region on the compression side, as well as nociceptive pain behavior in rats.
			• Pulse	Nickel titanium closed coil spring	
			• 560 mW		
			• 100.8 J/cm^2^		
			• 90 s		

## Data Availability

No new data were created or analyzed in this study. Data sharing is not applicable to this article.

## Abbreviations

The following abbreviations are used in this manuscript:

AKT	protein kinase B
ALP	alkaline phosphatase
ASIC3	acid-sensing ion channel 3
BMP	bone morphogenetic protein
CGRP	calcitonin gene-related peptide
CNS	central nervous system
COX-2	cyclooxygenase-2
DIV	days in vitro
ETM	experimental tooth movement
GFAP	glial fibrillary acidic protein
Iba-1	ionized calcium-binding adapter molecule-1
IFN-γ	interferon gamma
IL	interleukin
iNOS	inducible nitric oxide synthase
JAK2	Janus kinase 2
JOR	jaw-opening reflex
LED	light-emitting diode
LLLT	low-level laser therapy
M-CSF	macrophage colony-stimulating factor
Nd:YAG	neodymium-doped yttrium aluminum garnet
Nlgn3	neuroligin-3
OGD	oxygen–glucose deprivation
OPG	osteoprotegerin
PBM	photobiomodulation
PBMT	photobiomodulation therapy
PDL	periodontal ligament
PGE_2_	prostaglandin E_2_
POSTN	periostin
PSD95	postsynaptic density protein 95
P2X3	purinergic P2X receptor 3
RANKL	receptor activator of nuclear factor kappa B ligand
SOST	sclerostin
STAT3	signal transducer and activator of transcription 3
TGF-β	transforming growth factor beta
TIMPs	tissue inhibitors of metalloproteinases
TNF-α	tumor necrosis factor alpha
Vc	trigeminal subnucleus caudalis
VCM	vacuous chewing movement
